# High-intensity focused ultrasound for endometrial ablation in adenomyosis: a clinical study

**DOI:** 10.3389/fmed.2024.1332080

**Published:** 2024-03-21

**Authors:** Siyun Wu, Jun Liu, Xiaoshan Liu, Yanhua Han

**Affiliations:** Department of Obstetrics and Gynecology, Zhongshan City People’s Hospital, Zhongshan, China

**Keywords:** high-intensity focused ultrasound, magnetic resonance imaging, adenomyosis, endometrium, dysmenorrhea

## Abstract

**Objective:**

The present study aimed to investigate the clinical efficacy of endometrial ablation with high-intensity focused ultrasound (HIFU) for symptom relief in women with adenomyosis.

**Methods:**

Between July 2014 and July 2020, 167 patients with adenomyosis treated at the Zhongshan City People’s Hospital were enrolled in this study. Patients were divided into two groups according to patient aspirations: the control group, including patients who only underwent ablation of adenomyosis lesions (group A) and the treatment group, including patients who underwent removal of adenomyosis lesions and endometrial ablation (group B).

**Results:**

The reduced dysmenorrhea scores (visual analog scale) and menstrual volume scores (pictorial blood assessment chart) were measured before and after treatment. The scores were obtained by subtracting the postoperative scores from the preoperative scores and were compared to determine whether the symptoms had alleviated. Compared with the menstrual volume of group A, that in group B showed significant improvements. The average relief rates of dysmenorrhea in the two groups also showed significant improvement. However, the scores in group B showed a more significant improvement than those in group A.

**Conclusion:**

Therefore, our findings suggest that endometrial ablation using HIFU may be superior to conventional therapy with regard to alleviating the symptoms of increased menstruation in women with adenomyosis.

## 1 Introduction

Adenomyosis is a benign uterine disease characterized by invasion of the endometrial glands and stroma in the uterus. It is a very common gynecological disorder that affects people of reproductive age and compromises their quality of life. This condition is frequently the underlying cause of dysmenorrhea, menorrhagia, and urinary dysfunction and can impact fertility. The distinguishing factors of magnetic resonance imaging (MRI) in adenomyosis include a focal or uneven width of the junction zone, low signal intensity in the junctional zone, high signal spots on T2-weighted images scattered within the junctional zone during hemorrhage, and unclear zone margins (1).

Traditionally, adenomyosis has been treated medically or surgically. Currently, hysterectomy is the only curative treatment for adenomyosis. The boundary of the adenomyotic lesion is unclear, making it difficult to completely remove the lesions. Conservative surgery, i.e., adenomyomectomy, has proven to be effective only in approximately 50% of patients, and the recurrence rate is very high (2). In addition, there is no specific treatment for patients who wish to retain their uterus or remain fertile. Thus, it is important to explore effective, safe, and less invasive treatment strategies for these patients.

Uterus-conserving treatment of adenomyosis is a clinical challenge, and the margins of adenomyotic lesions are often ill-defined, posing difficulties for surgical resection. High-intensity focused ultrasound (HIFU), an emerging non-invasive treatment, features good tissue penetration and can induce coagulation necrosis of the targeted lesion. It does so through thermal and biological effects caused by the instant temperature increase from energy generated by ultrasound waves focused on the target within the body (3). Although it is difficult to ablate adenomyotic lesions completely using HIFU, this treatment causes little to no injury to the normal surrounding tissue. HIFU has been used to treat patients with symptomatic adenomyosis. Recently, several studies have investigated the role of HIFU in adenomyosis treatment; it has been shown to be safe and effective (4). However, most studies have focused on patients with dysmenorrhea, rather than on patients with menorrhagia. Therefore, the present study aimed to investigate the clinical efficacy of endometrial HIFU ablation for symptom relief in patients with adenomyosis.

## 2 Materials and methods

### 2.1 Patients

Between July 2014 and July 2020, 167 patients with adenomyosis were treated at Zhongshan City People’s Hospital and were enrolled in this study. The diagnosis of adenomyosis was confirmed using preprocedural MRI. All patients signed an informed consent form before HIFU treatment. Patients were divided into two groups according to the patients’ treatment goals: the control group (group A), only adenomyosis lesions ablated with HIFU, and treatment group (group B), adenomyosis lesions and endometrial ablation with HIFU. Patients undergoing endometrial ablation were likely to develop infertility; therefore, they were fully informed, and consent was obtained before joining the treatment group. Patients who wished to preserve fertility were assigned to group A.

The inclusion criteria were as follows: premenopausal adult women (age >18 years) presenting with clinical symptoms of dysmenorrhea and/or menorrhagia and agreed to undergo periodic checkups. The exclusion criteria were clinical examination or ultrasonography findings showing endometrial disease, pelvic endometriosis, or other uncontrolled systemic diseases, and menstruation, pregnancy, or lactation. Adenomyosis is divided into four types, including diffuse adenomyopathy, focal adenomyopathy, single/multiple adenomyoma (5). In this study, all four types of adenomyopathy were included.

### 2.2 HIFU ablation

HIFU was performed using an ultrasound-guided HIFU system (USgHIFU; JC200; Haifu Medical Technology Co., Ltd., Chongqing, China). The patients were preoperatively administered fentanyl and midazolam for sedation and analgesia. They were placed in the prone position on the HIFU treatment table to enable their skin to be in full contact with degassed water. A degassed water balloon was placed between the transducer and anterior abdominal wall to compress or push away the bowel. The treatment started from the center of the lesion as point sonication. The sonication power ranged from 350 to 400 W. During the procedure, the treatment area and sonication intensity were adjusted based on changes in the gray scale on ultrasound and patient tolerance. During treatment, patients were requested to lie still and report any discomfort, including burning of the skin or lower abdominal, leg, sciatic, or buttock pain (6).

### 2.3 Post-treatment examination

**Figure 1:**
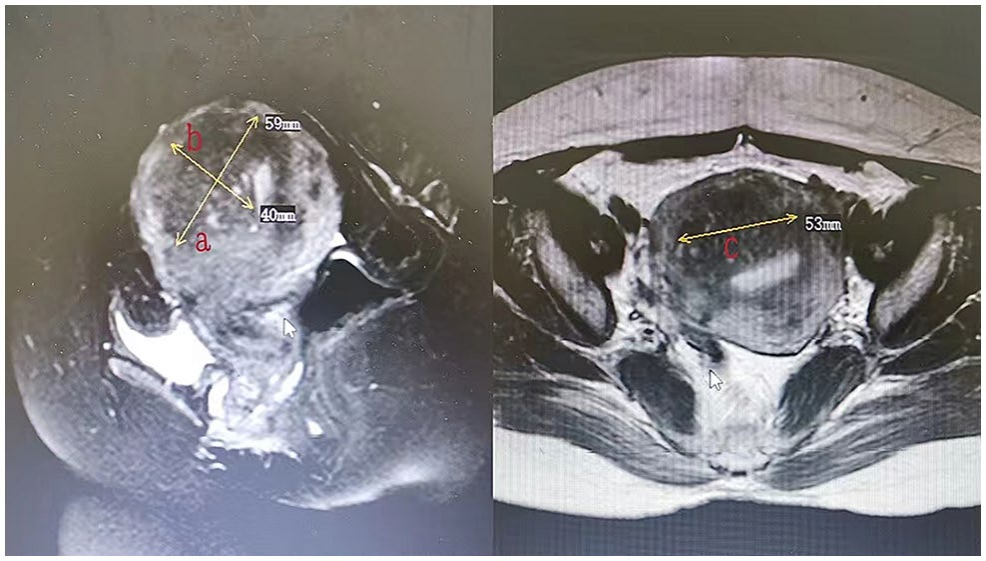
The measurement of the volume of adenomyotic lesions (volume = 0.5233 × *a* × *b* × *c*).

**Figure 2:**
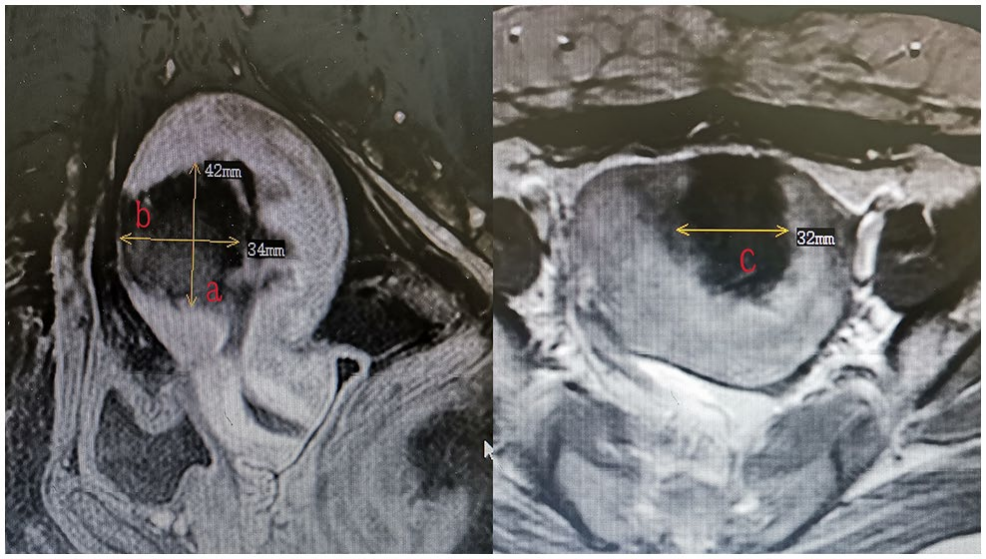
The measurement of the volume of the destroyed lesions (volume = 0.5233 × *a* × *b* × *c*).

All patients underwent MRI within 3 days after HIFU to define the condition of the endometrium and evaluate the non-perfused volume (NPV). The volume of adenomyotic lesions and NPV were measured using the following equation for the prolate ellipsoid (7): volume = 0.5233 × *a* × *b* × *c* (*a*, *b*, and *c* are the longitudinal, anteroposterior, and transverse dimensions, respectively). The volume of adenomyotic lesions was defined as the volume of the part of the uterus where the focal adenomyotic lesions were located (Figure 1). The volume of the ablation lesions was defined as the volume of the non-perfused area (Figure 2). The NPV ratio (NPVR) was defined as the NPV divided by the lesion volume, NPVR = NPV/volume of the adenomyotic lesion × 100%. Successful treatment with HIFU was defined as an NPV of ≥1 cm^3^ in the planned ablation zone (8).

**Figure 3:**
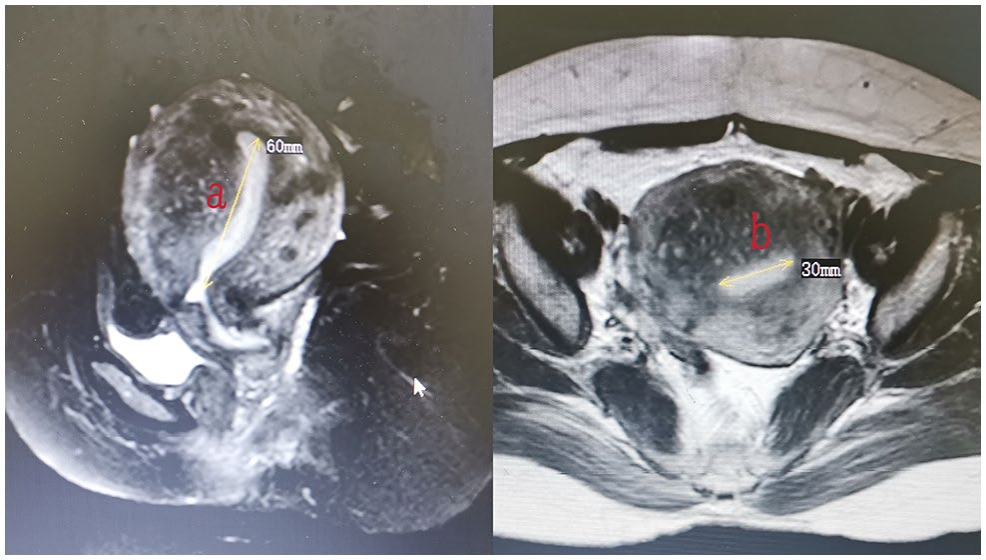
The measurement of the area of endometrium (area = 0.5 × *a* × *b* × 2).

**Figure 4:**
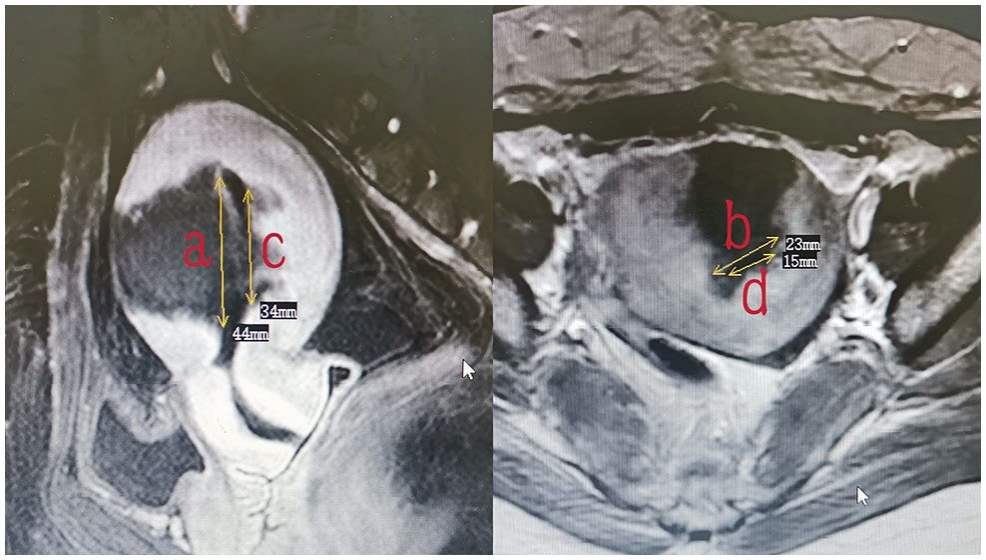
The measurement of the area of frustrated endometrium (area = 0.5 × *a* × *b* + 0.5 × *c* × *d*).

The endometrium of the uterine cavity is an inverted triangle, and the uterine cavity has two sides. The area of the endometrium was measured using the following equation: area = 0.5 × *a* × *b* (*a* and *b* are the longitudinal and transverse dimension, respectively) × 2. The measurements of the area of the intact endometrium and ablated endometrium were based on preoperative (Figure 3) and postoperative MRI findings (Figure 4), respectively. The ablation rate was defined as the area of frustrated endometrium divided by the area of endometrium. Ablation rate = (area of frustrated endometrium/area of endometrium) × 100%. Successful treatment with HIFU was determined as the occurrence of a destruction rate of ≥25%.

This study was approved by the Ethics Clerk Association of Zhongshan City People’s Hospital. All experiments were performed in accordance with relevant guidelines and regulations.

### 2.4 Evaluation of clinical symptoms after treatment

All adverse events and complications were recorded by a nurse during and after the procedure to examine the safety of HIFU. Follow-ups were performed by staff via telephone to register all post-surgical symptoms and complaints. There were two main observation indicators: dysmenorrhea and menstrual volume. The intensity of dysmenorrhea was assessed using a visual analog scale (VAS) score ranging from 0 to 10 (9). Menstrual volumes were scored according to the patients’ descriptions of pictorial blood assessment charts (10). The remission rate and clinical effectiveness were evaluated at 3, 6, and 12 months after HIFU ablation.

The reduced dysmenorrhea and menstrual volume scores before and after treatment, subtracted from the preoperative score by the postoperative score, were compared to determine whether the symptoms had alleviated. Symptoms were considered alleviated using the following criteria: (1) inefficient, with score reduction of 20%; (2) partial relief, with score reduction of 20–50%; (3) significant relief, with score reduction of 50–80%; and (4) complete relief, with score reduction of 80%. Clinical relief included partial, significant, and complete relief (11, 12, 13).

### 2.5 Statistical analysis

Data are presented as the mean ± standard deviation. SPSS (SPSSAU) was used for data analysis. The *t*-test was performed to compare the variables between the two groups. Statistical significance was set at a *p*-value of <0.05.

## 3 Results

### 3.1 Baseline patient characteristics

Overall, 167 patients with adenomyosis underwent ultrasound-guided HIFU (USgHIFU) ablation. Eligible patients were divided into two groups: the control group (69 patients) and treatment group (98 patients). The average age of the patients was 39.72 ± 5.49 years (group A) and 40.15 ± 5.38 years (group B), with no significant differences between the groups. All patients successfully underwent the complete procedure and treatment. The destruction rates of the endometrium were 0.36 ± 0.61% (group A) and 43.2 ± 21.1% (group B); the difference between the two groups was statistically significant (Table 1). Some patients in group A had endometrial ablation caused by poor energy control; however, this occurred in only a small number of patients, and the ablation rate was <5%. The VAS score in group A was 7.51 ± 1.26, compared with 8.32 ± 0.82 in group B. The score of menstrual volumes was 137.97 ± 42.59 in group A and 163.47 ± 30.19 in group B. The difference between both groups was significant for each measure.

**Table 1:** Baseline characteristics of patients.

	Group (mean ± SD)		*p*
	A (*n* = 69)	B (*n* = 98)	
Age (years)	39.72 ± 5.49	40.15 ± 5.38	0.616
Destruction rate of the endometrium (%)	0.36 ± 0.61	43.2 ± 21.1	0.00^**^
Pretreatment VAS	7.51 ± 1.26	8.32 ± 0.82	0.00^**^
Pretreatment menstrual volumes	137.97 ± 42.59	163.47 ± 30.19	0.00^**^

^**^p < 0.01.

### 3.2 Results of treatment

As shown in Table 2, the HIFU treatment settings and procedures displayed no significant difference in the time of treatment between patients in the groups. A significant difference was noted in the NPVR between the two groups: 69.77 ± 17.92% in group A and 81.63 ± 15.16% in group B.

**Table 2:** HIFU treatment results in patients from the two groups.

	Group (mean ± SD)		*p*
	A (*n* = 69)	B (*n* = 98)	
Time of treatment(s)	881.57 ± 458.01	874.48 ± 417.81	0.918
NPVR (%)	69.77 ± 17.92	81.63 ± 15.16	0.00^**^

^**^p < 0.01. HIFU, high-intensity focused ultrasound.

### 3.3 Symptom relief

The clinical efficacy rates of dysmenorrhea (score reduction of 20–100%) at 3, 6, and 12 months were 81.16, 86.96, and 85.50%, respectively, in group A and 95.91, 97.95, and 97.95%, respectively, in group B. There was a significant difference between the clinical efficacy rates of dysmenorrhea in the two groups (Table 3).

**Table 3:** Clinical effective rate of VAS in patients with dysmenorrhea after HIFU treatment.

Follow-up time	Group A (*n* = 69)	Group B (*n* = 98)	*p*
3 months	56 (81.16%)	94 (95.91%)	0.00^**^
6 months	60 (86.96%)	96 (97.95%)	0.00^**^
12 months	59 (85.50%)	96 (97.95%)	0.00^**^

^**^p < 0.01. VAS, visual analog scale; HIFU, high-intensity focused ultrasound.

As shown in Table 4, the clinical efficacy rates of menstrual volume (score reduction of 20–100%) at 3, 6 and 12 months were 24.63, 30.43, and 36.23%, respectively, in group A and 89.80, 95.91, and 94.80%, respectively, in group B. There was a significant difference between the clinical efficacy rates of menstrual volume in the two groups.

**Table 4:** Clinical effective rate of menstrual volumes in the patients after HIFU treatment.

Follow-up time	Group A (*n* = 69)	Group B (*n* = 98)	*p*
3 months	17 (24.63%)	88 (89.80%)	0.00^**^
6 months	21 (30.43%)	94 (95.91%)	0.00^**^
12 months	25 (36.23%)	93 (94.80%)	0.00^**^

^**^p < 0.01. HIFU, high-intensity focused ultrasound.

Table 5 shows the reduction in VAS score after HIFU treatment compared with that before treatment in both groups. The mean dysmenorrhea scores of patients from group A decreased by 3.23 ± 2.08, 3.59 ± 2.07, and 3.87 ± 2.31 points at 3, 6, and 12 months after treatment, respectively. The mean dysmenorrhea scores of patients from group B decreased by 5.03 ± 1.87, 5.55 ± 1.66, and 5.68 ± 1.70 points at 3, 6, and 12 months after treatment, respectively.

**Table 5:** Reduction of VAS in the patients after HIFU treatment.

Time	Group (mean ± SD)		*t*-value	*p*
	A (*n* = 69)	B (*n* = 98)		
3 months	3.23 ± 2.08	5.03 ± 1.87	5.729	0.000^**^
6 months	3.59 ± 2.07	5.55 ± 1.66	6.502	0.000^**^
12 months	3.87 ± 2.31	5.68 ± 1.70	5.559	0.000^**^

^**^p < 0.01. VAS, visual analog scale; HIFU, high-intensity focused ultrasound.

Table 6 shows the reduction in menstrual volume after HIFU treatment compared with that before treatment in both groups. The mean menstrual volumes of patients from group A decreased by 14.64 ± 26.63, 18.64 ± 28.77, and 20.87 ± 29.71 points at 3, 6, and 12 months after treatment, respectively. The mean menstrual volumes of patients from group B decreased by 69.52 ± 31.38, 83.35 ± 28.66, and 86.18 ± 28.30 points at 3, 6, and 12 months after treatment, respectively. There was a significant difference (*p* < 0.05) between the two groups in this regard. No complications occurred during the follow-up.

**Table 6:** Reduction of menstrual volumes in the patients after HIFU treatment.

Time	Group (mean ± SD)		*t*-value	*p*
	A (*n* = 69)	B (*n* = 98)		
3 months	14.64 ± 26.63	69.52 ± 31.38	11.833	0.000^**^
6 months	18.64 ± 28.77	83.35 ± 28.66	14.344	0.000^**^
12 months	20.87 ± 29.71	86.18 ± 28.30	14.385	0.000^**^

^**^p < 0.01. HIFU, high-intensity focused ultrasound.

## 4 Discussion

Adenomyosis is a common gynecological disease with an increasing prevalence (14). Several recent studies have demonstrated the safety and efficacy of HIFU ablation in the treatment of adenomyosis (15). However, most studies thus far have focused on dysmenorrhea and have ignored the symptoms of heavy menstruation.

**Figure 5:**
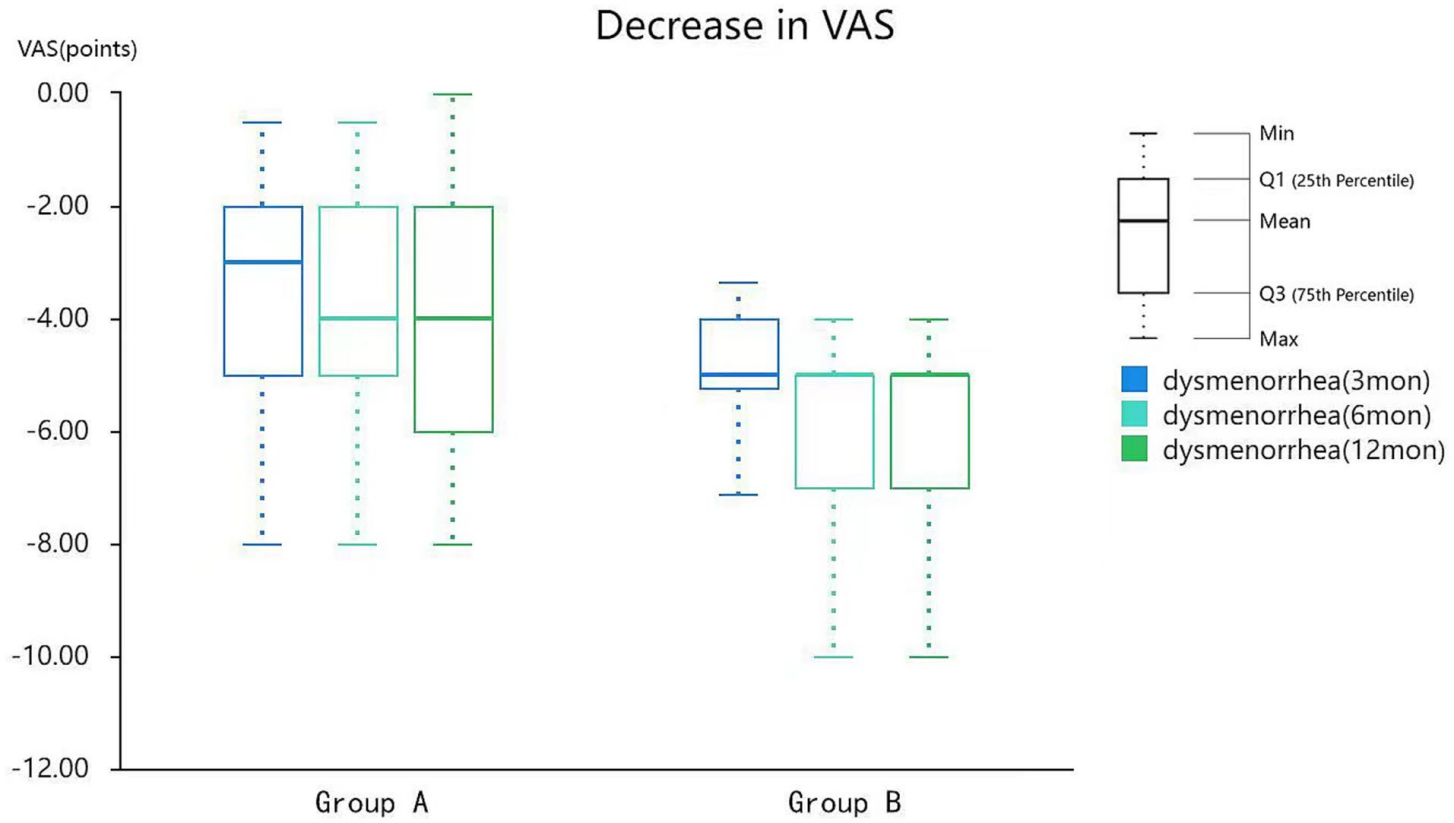
Reduction of VAS in patients after HIFU treatment. VAS, visual analog scale; HIFU, high-intensity focused ultrasound.

The relief rate of dysmenorrhea in our study at the 1 year follow-up after treatment was above 80%, consistent with that reported in a previous study (9). The dysmenorrhea score decreased significantly in the treatment group compared with that in the control group, indicating the effectiveness of the treatment (Figure 5). This finding may be related to the difference in NPVR between the two groups. Furthermore, this observation was highlighted in a regression analysis of factors affecting adenomyosis treatment published by Gong et al. (16). This study conducted a multivariate analysis of the effects of HIFU treatment for adenomyosis and found that NPVR was the most influential independent factor.

The common complications of HIFU treatment include skin burn, nerve injury, intestinal perforation, bladder perforation, etc. (17). The follow-up period in this study was 1 year after treatment, during which no patient required hysterectomy due to acute complications. Postoperative vaginal bleeding is a normal symptom of adenomyosis surgery, which usually lasts 2–4 weeks. This symptom was not included as a complication in the current study, so this study did not include this symptom as a complication follow-up.

**Figure 6:**
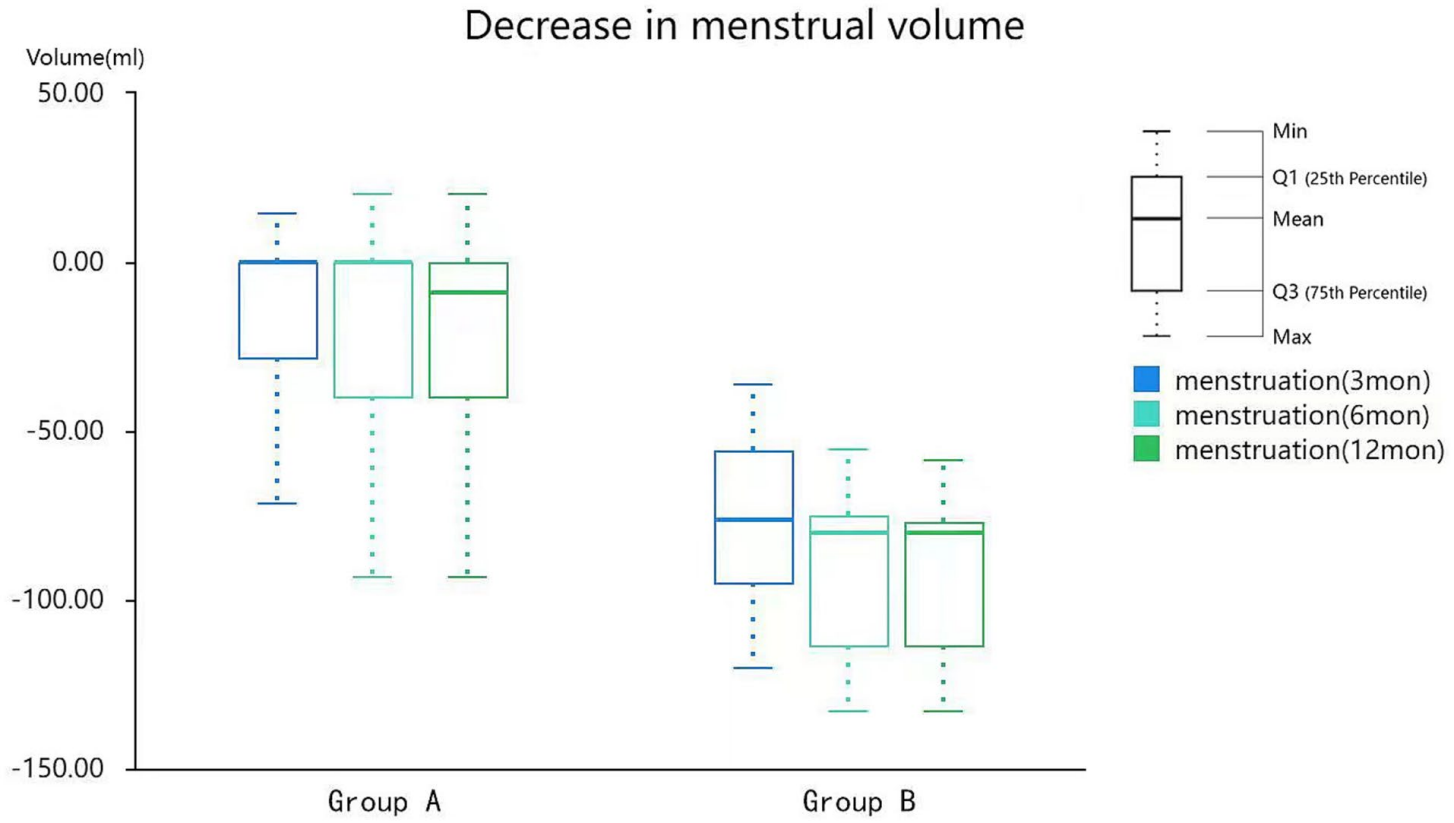
Reduction of menstrual volumes in patients after HIFU treatment. HIFU, high-intensity focused ultrasound.

Moreover, according to Guo et al. (18) the symptoms of menorrhagia can be ameliorated in patients with adenomyosis treated with HIFU alone. The patients in the control group in this study were also treated with HIFU alone, and the remission rate of menorrhagia symptoms was only 24–36% at the 1 year follow-up (Table 4); however, the remission rate in the treatment group was significantly higher (>80%). In addition, the improvement in menstrual scores before and after treatment in the two groups was compared with the declining value of the score. The decrease in the menstrual score in the treatment group was significantly greater than that in the control group (Figure 6), indicating that the menstrual volume in the treatment group significantly improved after endometrial ablation. A recent study (19) further showed that HIFU therapy can alleviate adenomyosis-associated menorrhagia to a small extent. The endometrial ablation method used in the present study was found to be a remedy for adenomyosis-associated menorrhagia. In addition, in this study, both groups had patients with symptoms that could not be improved at all, and group A had significantly more patients than group B. NPV in these patients did not differ significantly from patients whose symptoms improved, and we will find out why in future studies. In this study, the two groups of patients were not treated with gonadotropin-releasing hormone (GNRH) after HIFU. According to a study published by Yang et al. (20), GNRH administration combined with HIFU can control menstrual volume more effectively after ablation for adenomyosis. Levonorgestrel intrauterine birth control system is also an effective measure to relieve the symptoms of adenomyosis (21). However, the present study aimed to evaluate the effect of endometrial ablation on menstrual volume control; therefore, GNRH and IUD were not combined with treatment. Clinical studies assessing the effects of GNRH/IUD administration combined with ablation of the endometrium can be conducted in the future to clarify the relationship between these two modalities with regard to treating adenomyosis.

In the present study, endometrial ablation was performed via HIFU in patients with adenomyosis to observe its therapeutic effects. The results showed that the therapeutic effects of this treatment were better than those in patients not subjected to endometrial ablation.

However, this study had a few limitations. First, the sample size was limited, and this study was a non-randomized controlled trial. Second, the original scores of the two groups in this study were different. Although our evaluation indicators were compared by self-improvement scores, there was still some bias. Third, some symptomatic patients in both groups experienced no relief at all; this may be related to the ablation rate. Patients with a high ablation rate had a correspondingly high rate of symptom relief compared with those with a low ablation rate. Further studies should be conducted to validate these findings.

In conclusion, endometrial ablation is more effective than conventional therapy for the treatment of adenomyosis with regard to the alleviation of the symptoms of increased menstruation. Both treatments were effective in ameliorating menstrual cramps, but endometrial ablation was preferable, provided the patient does not wish to remain fertile.

## Data availability statement

The raw data supporting the conclusions of this article will be made available by the authors, without undue reservation.

## Ethics statement

The studies involving humans were approved by Ethics Committee of Zhongshan City People’s Hospital. The studies were conducted in accordance with the local legislation and institutional requirements. The participants provided their written informed consent to participate in this study.

## Author contributions

SW: Conceptualization, Data curation, Formal analysis, Writing – original draft. JL: Data curation, Funding acquisition, Writing – original draft. XL: Data curation, Writing – original draft. YH: Writing – review & editing.

## References

[1] Sudderuddin S Helbren E Telesca M Williamson R Rockall A. MRI appearances of benign uterine disease. Clin Radiol. (2014) 69:1095–104. doi: 10.1016/j.crad.2014.05.108 25017452

[2] Shrestha A Shrestha R Sedhai LB Pandit U. Adenomyosis at hysterectomy: prevalence, patient characteristics, clinical profile and histopatholgical findings. Kathmandu Univ Med J. (2012) 10:53–6. doi: 10.3126/kumj.v10i1.6915, PMID: 22971863

[3] Tsai MC Chang LT Tam KW. Comparison of high-intensity focused ultrasound and conventional surgery for patients with uterine myomas: a systematic review and meta-analysis. J Minim Invasive Gynecol. (2021) 28:1712–24. doi: 10.1016/j.jmig.2021.06.002, PMID: 34126271

[4] Zhang L Rao F Setzen R. High intensity focused ultrasound for the treatment of adenomyosis: selection criteria, efficacy, safety and fertility. Acta Obstet Gynecol Scand. (2017) 96:707–14. doi: 10.1111/aogs.13159, PMID: 28437003

[5] Kishi Y Suginami H Kuramori R Yabuta M Suginami R Taniguchi F. Four subtypes of adenomyosis assessed by magnetic resonance imaging and their specification. Am J Obstet Gynecol. (2012) 207:114.e1-7. doi: 10.1016/j.ajog.2012.06.027, PMID: 22840719

[6] Fu X Huang F Chen Y Deng Y Wang Z. Application of dexmedetomidine-remifentanil in high-intensity ultrasound ablation of uterine fibroids: a randomised study. BJOG. (2017) 124:23–9. doi: 10.1111/1471-0528.14740, PMID: 28856857

[7] Peng S Zhang L Hu L Chen J Ju J Wang X. Factors influencing the dosimetry for high-intensity focused ultrasound ablation of uterine fibroids: a retrospective study. Medicine. (2015) 94:e650. doi: 10.1097/MD.0000000000000650, PMID: 25837756 PMC4554030

[8] Ahmed M Solbiati L Brace CL Breen DJ Callstrom MR Charboneau JW. Image-guided tumor ablation: standardization of terminology and reporting criteria—a 10-year update. J Vasc Interv Radiol. (2014) 25:1691–705.e4. doi: 10.1016/j.jvir.2014.08.027, PMID: 25442132 PMC7660986

[9] Liu X Wang W Wang Y Wang Y Li Q Tang J. Clinical predictors of long-term success in ultrasound-guided high-intensity focused ultrasound ablation treatment for adenomyosis: a retrospective study. Medicine. (2016) 95:e2443. doi: 10.1097/MD.0000000000002443, PMID: 26817877 PMC4998251

[10] Higham JM O’Brien PM Shaw RW. Assessment of menstrual blood loss using a pictorial chart. Br J Obstet Gynaecol. (1990) 97:734–9. doi: 10.1111/j.1471-0528.1990.tb16249.x 2400752

[11] Goodwin SC Bonilla SC Sacks D Reed RA Spies JB Landow WJ. Reporting standards for uterine artery embolization for the treatment of uterine leiomyomata. J Vasc Interv Radiol. (2003) 14:S467–76. doi: 10.1097/01.RVI.0000094620.61428.9c 14514862

[12] Zhang X Li K Xie B He M He J Zhang L. Effective ablation therapy of adenomyosis with ultrasound-guided high-intensity focused ultrasound. Int J Gynaecol Obstet. (2014) 124:207–11. doi: 10.1016/j.ijgo.2013.08.022 24380611

[13] Shui L Mao S Wu Q Huang G Wang J Zhang R. High-intensity focused ultrasound (HIFU) for adenomyosis: two-year follow-up results. Ultrason Sonochem. (2015) 27:677–81. doi: 10.1016/j.ultsonch.2015.05.024, PMID: 26050604

[14] Moawad G Kheil MH Ayoubi JM Klebanoff JS Rahman S Sharara FI. Adenomyosis and infertility. J Assist Reprod Genet. (2022) 39:1027–31. doi: 10.1007/s10815-022-02476-2, PMID: 35347501 PMC9107544

[15] Marques ALS Andres MP Kho RM Abrão MS. Is high-intensity focused ultrasound (HIFU) effective for the treatment of adenomyosis? A systematic review and meta-analysis. J Minim Invasive Gynecol. (2020) 27:332–43. doi: 10.1016/j.jmig.2019.07.029, PMID: 31377454

[16] Gong C Yang B Shi Y Liu Z Wan L Zhang H. Factors influencing the ablative efficiency of high intensity focused ultrasound (HIFU) treatment for adenomyosis: a retrospective study. Int J Hyperth. (2016) 32:496–503. doi: 10.3109/02656736.2016.1149232, PMID: 27385316

[17] Zhou M Chen JY Tang LD Chen WZ Wang ZB. Ultrasound-guided high-intensity focused ultrasound ablation for adenomyosis: the clinical experience of a single center. Fertil Steril. (2011) 95:900–5. doi: 10.1016/j.fertnstert.2010.10.020, PMID: 21067723

[18] Guo Y Duan H Cheng J Zhang Y. Gonadotrophin-releasing hormone agonist combined with high-intensity focused ultrasound ablation for adenomyosis: a clinical study. BJOG. (2017) 124:7–11. doi: 10.1111/1471-0528.14736, PMID: 28856862

[19] Li W Mao J Liu Y Zhu Y Li X Zhang Z. Clinical effectiveness and potential long-term benefits of high-intensity focused ultrasound therapy for patients with adenomyosis. J Int Med Res. (2020) 48:300060520976492. doi: 10.1177/0300060520976492, PMID: 33349096 PMC7758569

[20] Yang X Zhang XF Lin B Feng X Aili A. Combined therapeutic effects of HIFU, GnRH-a and LNG-IUS for the treatment of severe adenomyosis. Int J Hyperth. (2019) 36:485–91. doi: 10.1080/02656736.2019.1595179, PMID: 30994010

[21] Moawad G Youssef Y Fruscalzo A Faysal H Kheil M Pirtea P. The present and the future of medical therapies for adenomyosis: a narrative review. J Clin Med. (2023) 12:6130. doi: 10.3390/jcm12196130, PMID: 37834773 PMC10573655

